# A graph-based algorithm for detecting rigid domains in protein structures

**DOI:** 10.1186/s12859-021-03966-3

**Published:** 2021-02-12

**Authors:** Truong Khanh Linh Dang, Thach Nguyen, Michael Habeck, Mehmet Gültas, Stephan Waack

**Affiliations:** 1 Institute of Computer Science, University of Göttingen, Goldschmidtstr 7, 37077 Göttingen, Germany; 2 Felix Bernstein Institute for Mathematical Statistics in the Biosciences, University of Göttingen, Goldschmidtstr 7, 37077 Göttingen, Germany; 3 Max Planck Institute for Biophysical Chemistry, Am Fassberg 11, 37077 Göttingen, Germany; 4 Microscopic Image Analysis Group, University Hospital Jena, Am Klinikum 1, 07747 Jena, Germany; 5 Breeding Informatics Group, Department of Animal Sciences, Margarethe von Wrangell-Weg 7, 37075 Göttingen, Germany; 6 Center for Integrated Breeding Research (CiBreed), Albrecht-Thaer-Weg 3, 37075 Göttingen, Germany

**Keywords:** Protein structural transition, Graph algorithms, Generalized Viterbi algorithm

## Abstract

**Background:**

Conformational transitions are implicated in the biological function of many proteins. Structural changes in proteins can be described approximately as the relative movement of rigid domains against each other. Despite previous efforts, there is a need to develop new domain segmentation algorithms that are capable of analysing the entire structure database efficiently and do not require the choice of protein-dependent tuning parameters such as the number of rigid domains.

**Results:**

We develop a graph-based method for detecting rigid domains in proteins. Structural information from multiple conformational states is represented by a graph whose nodes correspond to amino acids. Graph clustering algorithms allow us to reduce the graph and run the Viterbi algorithm on the associated line graph to obtain a segmentation of the input structures into rigid domains. In contrast to many alternative methods, our approach does not require knowledge about the number of rigid domains. Moreover, we identified default values for the algorithmic parameters that are suitable for a large number of conformational ensembles. We test our algorithm on examples from the DynDom database and illustrate our method on various challenging systems whose structural transitions have been studied extensively.

**Conclusions:**

The results strongly suggest that our graph-based algorithm forms a novel framework to characterize structural transitions in proteins via detecting their rigid domains. The web server is available at http://azifi.tz.agrar.uni-goettingen.de/webservice/.

## Background

Proteins are molecular machines that are involved in a large variety of biological processes. Protein function is often driven by large-scale structural transitions [1]. Experimental methods for biomolecular structure determination such as X-ray crystallography, NMR and cryo-electron microscopy have been used to determine thousands of atomic structures of proteins in different conformational states. A powerful approach to understand structural transitions in proteins is to decompose structures of different states into rigid domains and classify protein movements by hinge and shear motions of these structural domains [2].

Given the large number of available protein structures, we need computational methods that identify structurally conserved domains in a set of alternative structures in an automated fashion with minimal user intervention. For example, one could use the software to study molecular dynamics trajectories at the level of rigid domains to gain an understanding of large-scale movements, or identify important active sites located at the interface between rigid domains.

A number of computational methods for detecting rigid domains in protein structures have been developed. Dyndom [3] identifies rigid domains by clustering a set of rotation vectors. Hingefind [4] focuses on the detection of hinge residues, which are detected via differences in bending angles. RigidFinder [5] finds rigid domains via a dynamic programming algorithm that optimizes the rigidity of structural segments extracted from two conformational states. These methods are limited to two input structures and require the selection of a cutoff parameter [5], which can impact the results quite strongly. Spectrus [6] applies spectral clustering to distance fluctuations and supports multiple input structures. However, the number of clusters relies on a quality score, which sometimes gives ambiguous results. Probabilistic approaches [7, 8] segment protein structures into rigid domains as part of a generative probabilistic model. The model parameters, including the segmentation, are inferred with expectation maximization or Gibbs sampling. However, choosing the initial parameters as well as the number of rigid segments is still a critical issue, because both algorithms explore parameter space only locally, and can therefore require many restarts from different initial conditions.

A more ambitious goal is to predict rigid domains from a single structure by, for example, molecular dynamic simulation or an elastic network model that can both be used to generate a set of alternative conformational states. HingeProt [9] and Domain Finder [10] use an elastic network model to predict hinge residues by analyzing the correlation between selected pairs of eigenvectors of the correlation matrix. However, in general it is unclear which modes contribute most strongly to the movement, in particular if a conformational change involves multiple modes. FlexOracle [11] finds hinge positions by identifying split points with minimal energetic impact.

Despite the rich literature on methods for rigid-domain detection in protein structures, all of the existing methods require the initial number of rigid domains in their calculation. Thus, there is still a need for algorithms that are robust, reliable, able to handle high-throughput data and yet do not require extensive parameter tunning. Here, we introduce a graph-based method that infers a binary labeling that encodes if pairs of amino acids belong to identical or different rigid domains. Our algorithm proceeds in two stages: first, we construct a protein graph based on spatial proximity, which we cluster using the Louvain algorithm to obtain a coarse-grained graph of reduced size. Second, edges in the reduced graph are labeled by applying a line graph transformation along with the general Viterbi algorithm. We benchmark our algorithm on 487 entries of the DynDom database and find a high agreement with the reference segmentation. In addition, we also present a detailed analysis of various proteins that show a large variety of conformational transitions and compare our results to other methods.

**Fig. 1:**
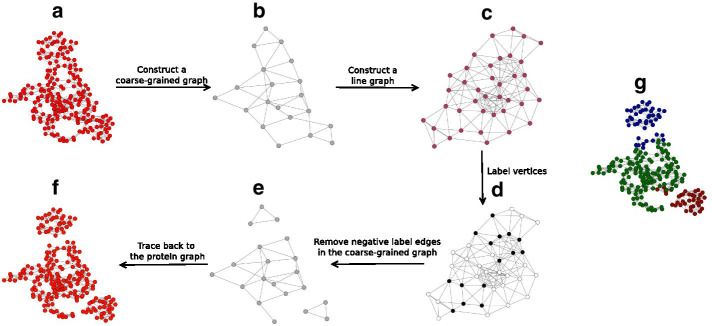
Graph-based segmentation of ADK into rigid domains. **a** Protein graph constructed from open and closed conformations. **b** Reduced graph obtained by coarse-graining the protein graph. **c** A line graph of the reduced graph. **d** A line graph with binary vertex labels (black: − 1, white: + 1) obtained with the generalized Viterbi algorithm. **e** The injective relation between edges of the reduced graph and vertices of the line graph allows us to also label the edges of the reduced graph. Edges having negative labels are removed resulting in three disconnected subgraphs. **f** A segmented protein graph derived from disconnected subgraphs in the reduced graph. **g** ADK graph with domain annotation from literature encoded by colors

## Results

To validate our algorithm, we first segment conformations of Adelynate Kinase (ADK). We then perform a benchmark on 487 proteins from the DynDom database. Finally, we compare our method with other domain segmentation algorithms on a number of test cases ranging from medium to large scale conformational changes.

### Rigid segmentation of Adenylate Kinase

We first run our algorithm for rigid domain segmentation on Adenylate Kinase (ADK) for which multiple experimental structures showing different conformations are available [12]. ADK catalyzes the interconversion of adenine nucleotides and is composed of three rigid domains. By closing the NMP-binding domain and the LID domain onto the CORE domain, ADK binds ATP and AMP which are converted to two ADP molecules. The PDB codes of ADK open and closed conformations are 4ake and 1ake (both chain A) respectively. ADK is composed of 214 amino acids which constitute the vertices of the initial protein graph. To build the protein graph from both states, we used $\delta =7.5$ Å as cutoff.

Figure 1 illustrates the workflow of our algorithm and intermediate results for ADK using default values for the algorithmic parameters. Figure 1a shows ADK’s protein graph in which each vertex is an amino acid; the construction of edges linking spatially close amino acids is described in Methods. Amino acids are grouped by running the Louvain domain detection algorithm [13] and merged into vertices of a coarse-grained graph. In the case of ADK, the protein graph comprising 214 vertices is transformed to a coarse-grained graph composed of 20 vertices (Fig. 1b). In the next step, we construct the line graph of the coarse-grained graph (Fig. 1c). We then run the generalized Viterbi algorithm [14] on a scoring function defined on the line graph. This results in a binary labeling of the line graph (Fig. 1d) or, equivalently, a labeling of the coarse-grained graph. Based on this labeling our method splits the coarse-grained graph into three disconnected subgraphs (Fig. 1e). Finally, we map the unconnected subgraphs back to the protein graph to obtain a segmentation of ADK into three rigid domains (Fig. 1f). Our segmentation agrees strongly with the domain boundaries defined in the literature [15], which we color-coded in Fig. 1g for visual comparison. Our segmentation deviates from the literature annotation only in the hinge regions. This discrepancy is due to the ambiguous membership of amino acids in the hinge region which tend to be merged with amino acids from different domains in the coarse-graining step.

Unlike DynDom, our method also works with multiple conformational states. To study this feature, we ran our algorithm again but on 100 ADK conformations generated by morphing between the open and closed state [16]. The algorithm produces a similar segmentation.

An advantage of our method is that it allows users to integrate prior knowledge to improve the segmentation. For example, for the default parameter setting, our method incorrectly assigned fifteen amino acids of the NMP-binding and LID domain to the core domain. Yet with some prior knowledge about the rigid domains, we can improve the rigid-domain segmentation. Suppose we are given ADK’s segmentation calculated from Spectrus [6] with $K=4$ (number of rigid domains). We can integrate this prior knowledge into our model as follows. The weights of edges in the protein graph whose vertices belong to different domains according to the prior labeling are reduced by a factor $\alpha <1$. Here, we choose $\alpha = 0.75$. This setting helps the coarse-graining process to reduce the error of inconsistency (mentioned in the Discussion) and thus improve the performance. We then ran our graph-based method on the new coarse-grained graph and found that only five amino acids of the LID domain were wrongly assigned to the core domain. Thus even imperfect prior knowledge can significantly improve the result.

**Fig. 2:**
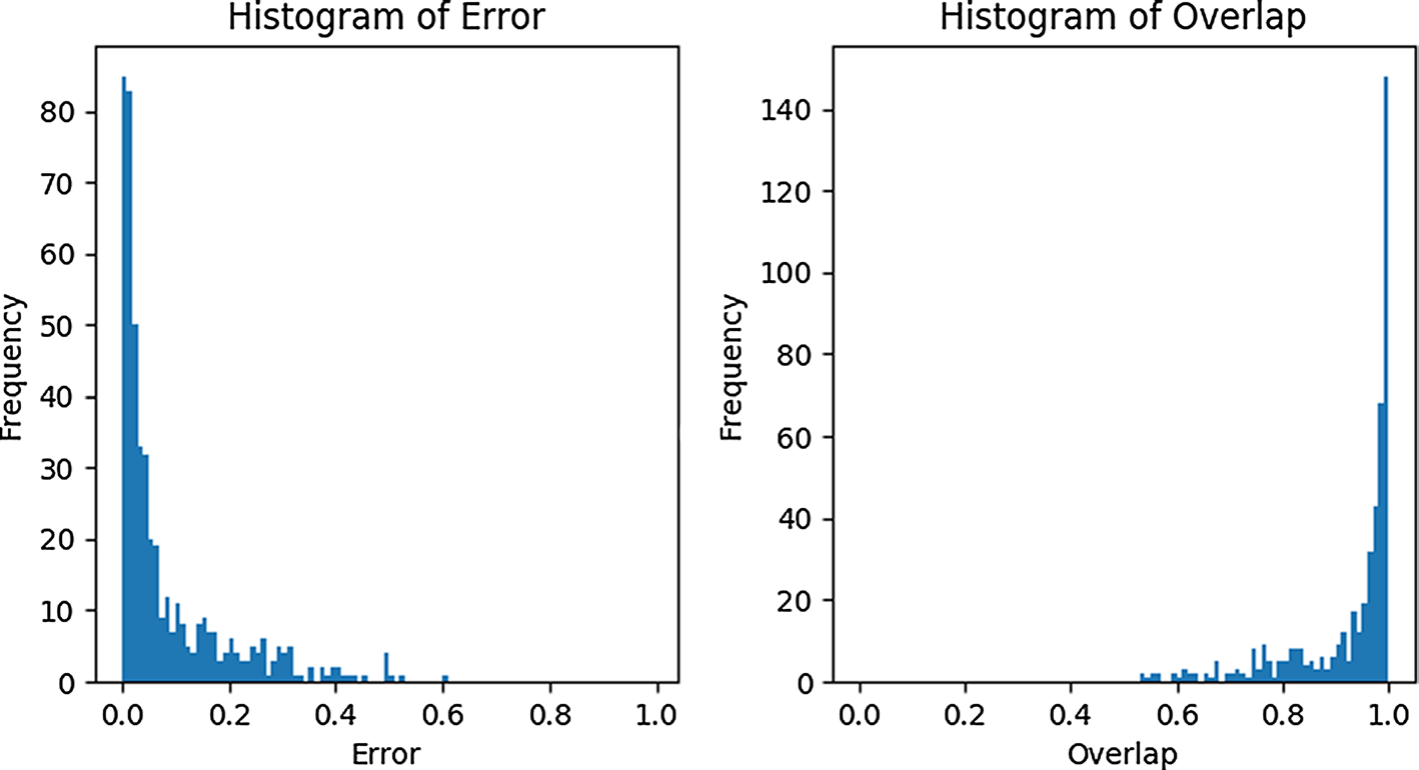
Histogram of the error and the overlap evaluated on 487 proteins in the DynDom database

### Rigid segmentation benchmark

We benchmarked our method on the DynDom database [17] reduced to those pairs of proteins whose overall RMSD exceeds 5 Å. Moreover, we removed domains that span less than ten amino acids. To evaluate our method, we use the segmentation error and overlap defined by [8]. The overlap counts the number of matches between two segmentations after solving a low-dimensional linear assignment problem that maximizes the agreement between the two labelings. The error assesses how often two segmentations disagree on whether a pair of amino acids belongs to the same domain. Although both metrics differ in the details, they are highly anti-correlated.

**Fig. 3:**
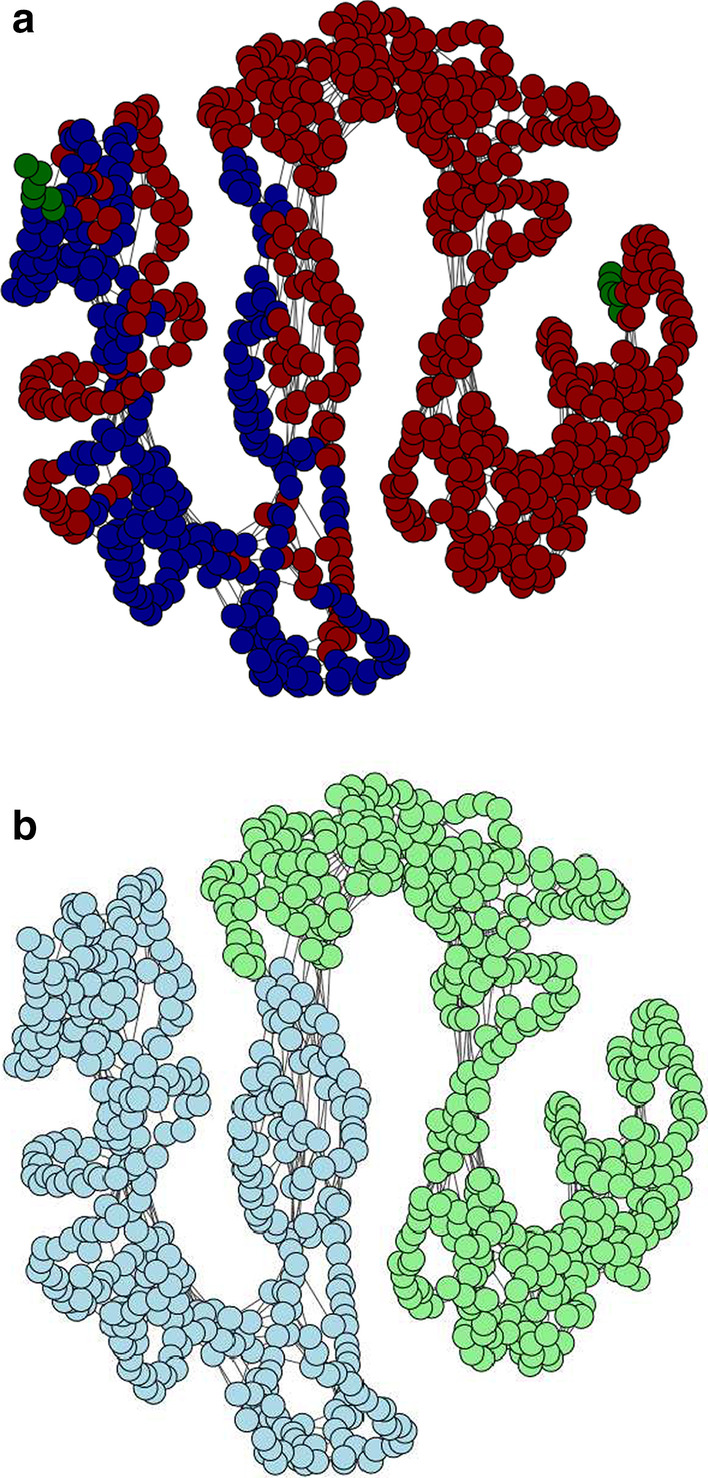
Protein graph of human importin subunit beta-1 protein. **a** Segmentation suggested by DynDom: three rigid domains colored in dark green, red and blue. **b** Segmentation estimated by our method: two rigid domains colored in light green and blue

Figure 2 shows histograms of the error and overlap between our and DynDom’s segmentation evaluated on 487 proteins based on an edge cutoff value of 7.5 Å. The median error is 0.038 and the median overlap 0.972. The error and overlap histograms are highly skewed to small and large values, respectively. For approximately 30% of the examples, our method reaches a near perfect agreement with the annotation provided by DynDom (overlap $\ge$ 0.99). In only a few cases our method fails to produce a reasonable segmentation due to errors in the coarse-graining step and/or an indistinguishable signal derived from the mean variance. Despite of the disagreements between our method and DynDom, our segmentation sometimes seems to be more reasonable. We investigate the open and closed states of human importin subunit beta-1 (PDB code 3lww, chains A and C) as an example. According to Dyndom, this protein has three rigid domains (Fig. 3a) whose ${\text{RMSD}}$s are 6.8, 4.3, and 2.1 Å, respectively. We note that the first domain found by DynDom (dark green) is small, fragmented and shows a large ${\text{RMSD}}$. A large portion of the second domain (dark red) is interspersed with the third domain (dark blue). Our segmentation suggests two separate domains whose ${\text{RMSD}}$s are 2.2 and 1.0 Å (Fig. 3b), which are much smaller than the RMSDs produced by DynDom’s segmentation.

To study the impact of the edge cutoff used in the definition of the protein graph, we ran experiments with varying cutoff values. Table 1 reports the mean and median of the overlap and error obtained with different edge cutoff values. The overlap seems to be largely unaffected by the specific choice of the cutoff, whereas the error drops slightly with larger cutoffs. Two possible explanations come to our mind. First, a larger cutoff results in protein graphs with more connections between amino acid vertices. Denser graphs seem to be more suitable to coarse graining with the Louvain method (see Additional file 1: Figure S1 and the Discussion for a demonstration of this claim). Second, also the coarse-grained graph will be denser with larger cutoff values, which seems to improve the scoring of the line graph. However, because denser graphs result in larger line graphs, we need to restrict the cutoff to smaller values to tame the computational costs of the Viterbi algorithm.

**Table 1:** Performance of the graph-based algorithm for different edge cutoffs evaluated on the DynDom benchmark

Cutoff (Å)	Metric			
	Median overlap	Mean overlap	Median error	Mean error
7.5	0.972	0.924	0.038	0.086
10.5	0.977	0.924	0.034	0.083
13.5	0.972	0.926	0.033	0.081

### Analysis of various structural transitions

**Fig. 4:**
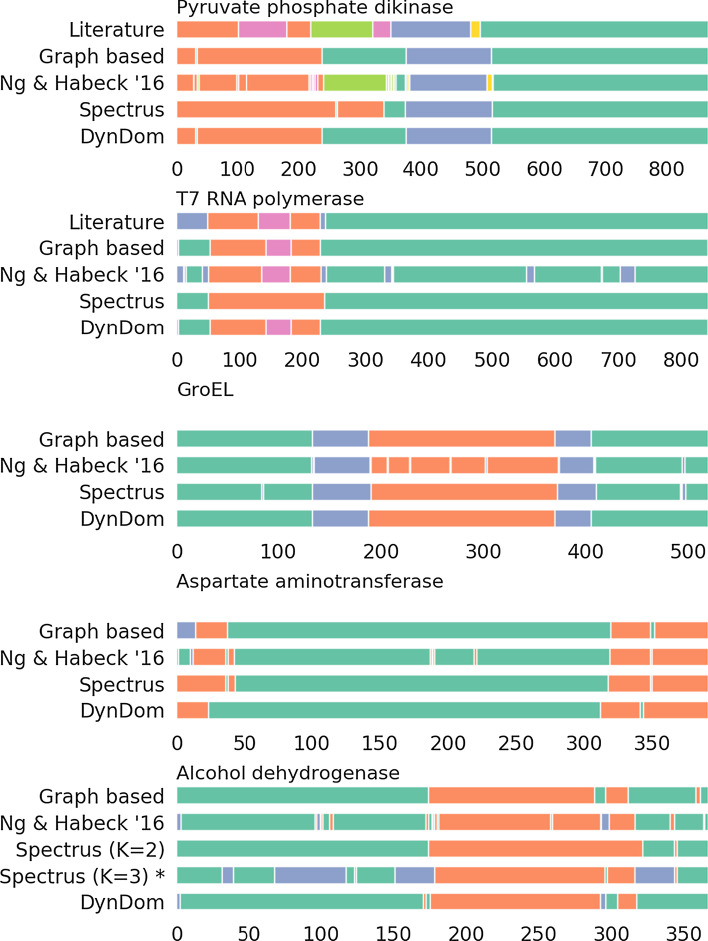
Analysis of several proteins undergoing conformational changes on a variety of scales. Large-scale conformational changes: pyruvate phosphate dikinase, T7 RNA polymerase, GroEL. Medium-scale conformational changes: Aspartate aminotransferase, Alcohol dehydrogenase. For each protein, the segmentation found by different methods and in the literature are shown. Same color means same domain

We ran our method on various proteins studied in [8] showing different types and scales of conformational changes. Table 2 provides the protein name, size and PDB code; Fig. 4 shows a summary of the segmentation analysis. First, we study and compare the performance of our algorithm (graph-based method) to other methods by analyzing protein complexes that undergo large-scale conformational changes.

Pyruvate phosphate dikinase (PPDK) is a large biomolecular complex that catalyzes the reversible conversion of PEP, AMP, and $\mathrm {P}_{\mathrm{i}}$ to pyruvate and ATP [18]. We apply our graph-based method to two PPDK structures and compare the segmentation to the annotation found in the literature [18] and by other methods such as Spectrus, DynDom as well as Nguyen&Habeck2016 [8]. Our segmentation agrees strongly with the segmentation provided by DynDom, but fails to detect the additional domain reported in the literature and by [8]. Typically, our method produces a smaller number of domains than reported in the literature, because we only take changes in a few structural snapshots into account and no additional experimental information. For $K=3$, Spectrus agrees strongly with the segmentation found by our graph-based approach except for the first domain, which is significantly larger according to Spectrus.

T7 RNA polymerase is involved in the initiation and elongation of RNA transcription. Our segmentation is highly consistent with the results from DynDom, [8] and the anotation from the literature [19]. Spectrus fails to identify the refolding loop inserted in the N-terminal domain.

The chaperonin GroEL [20] provides a shielded environment to assist protein folding and prevent aggregation. For this example, all methods provide very similar segmentation results.

We also benchmark our method on proteins undergoing medium-scale structural transitions. Aspartate aminotransferase (AST) is an enzyme involved in amino acid metabolism that catalyzes the reversible transfer of an $\alpha$-amino group between aspartate and glutamate [21]. For this example, we find a high agreement between our method and other segmentations. Another example is the enzyme Alcohol dehydrogenase (AhD) that decomposes alcohol into aldehyde. Our graph-based segmentation agrees strongly with the result from DynDom. Spectrus achieves its maximum score for $K=3$ domains, but introduces an additional domain compared to the other methods. For $K=2$, the score is lower, but Spectrus’ segmentation is more consistent to DynDom and our result.

**Table 2:** Proteins in different scale conformational changes involved in the assessment

Protein	PDB code	Chain ID	Size
PPDK	1kc7	A	872
	2r82	A	
T7 RNA polymerase	1qln	A	842
	1msw	D	
GroEL	1aon	A	524
	1aon	H	
Aspatate aminotransferase	9aat	A	401
	1ama	A	
Alcohol dehydrogenase	1adg	A	374
	2ohx	A	

## Discussion

Our results demonstrate that segmentation of protein conformations into rigid domains can be achieved with a graph-based algorithm that solves the rigid segmentation problem with an edge-labeling strategy. Let us discuss the key features of the algorithm and the impact of algorithmic parameters. To measure the efficiency of the graph construction and coarse graining, we use a metric that we call inconsistency error. The inconsistency error quantifies the heterogeneity of clusters weighted by their size. Let ${\mathcal {G}} = ({\mathcal {V}}, {\mathcal {E}})$ be a graph composed of $N=|{\mathcal {V}}|$ vertices $v_i \in {\mathcal {V}}$ with labels $\sigma _i$ and ${\mathcal {C}}=\{ {\mathcal {C}}_{k}\}$ a partition of the vertices into clusters ${\mathcal {C}}_{k} \subset {\mathcal {V}}$ obtained by coarse graining. We define the inconsistency error of the coarse graining procedure as $\mathrm {error}({\mathcal {C}} | {\mathcal {G}}) = 2 \sum _{{\mathcal {C}}_k \in C} \frac{|{\mathcal {C}}_k|}{N} \frac{\sum _{i<j \in {\mathcal {C}}_k} |\sigma _i \ne \sigma _j |}{|{\mathcal {C}}_k|\, (|{\mathcal {C}}_k|-1)}$ which is the average number of labeling mismatches within each cluster weighted by cluster size.

We first study different ways to construct a protein graph from multiple conformations. There are many reasonable options for constructing a protein graph. For example, one possibility is to create an edge if the distance between two vertices is smaller than a cutoff in at least one conformation, and to assign as a weight the number of such conformations. Another possibility (detailed in Methods) is to create an edge if its distance is smaller than the cutoff in *all* conformations, and to weight the edge by the reciprocal exponentiated variance computed over all conformations (such that low-variance edges have a weight close to one and large-variance edges are assigned small weights). Additional file 1: Figure S1 demonstrates that the second graph construction rule consistently outperforms the first rule based on the inconsistency error. We therefore used the second rule in our benchmark calculations. In addition, we tested different values of the edge cutoff distance and noticed a minor, but not significant improvement of the inconsistency error for larger cutoff values.

We also studied various options for the coarse-graining step. In all tests, we used the Louvain algorithm for fitting Potts models [13] for coarse graining. The resolution parameter was adjusted so as to produce about 20 clusters of medium size. Too large clusters risk to merge amino acids from hinge regions and thus the inconsistency error is expected to increase. Too small clusters will tend to show a smaller inconsistency error at the cost of lowering the significance of the mean variance between two clusters. Large graphs will pose a computational challenge in the Viterbi step, because the number of vertices of the line graph grows quadratically with the number of vertices in the original graph. By using our coarse-graining strategy, we save computational resources and enhance the signal as shown in Additional file 1 (see second section and Additional file 1: Figure S2).

Moreover, we ran our algorithm on Lysozyme [22], an enzyme contributing to the innate immune system, to investigate if this graph-based algorithm could produce a reasonable segmentation given several actual conformations. In this study, we use 100 conformations of Lysozyme whose PDB codes can be found in the Supplementary Information. To account for minor differences in the protein sequences, we align all proteins with Clustal Omega Alignment (https://www.ebi.ac.uk/Tools/msa/clustalo/). Our segmentation on Lysozyme completely agrees with Spectrus [6] and Nguyen&Habeck2016 [8] where all methods suggest two domains whose *RMSD*s are 1.6 and 4.9 Å, respectively.

Our method is also applicable to study rigid domains in membrane proteins. For instance, the chemokine receptor CCR5 [23] located on the surface of white blood cells plays an important role in the immune system. Here, we consider various conformational states of CCR5 (PDB codes: 6aky_A, 4mbs_A, 6akx_A, 5uiw_A). The sequences of these four conformational states were aligned with Clustal Omega [24, 25]. Our segmentation finds a small (51 amino acids 223–253) and a big (286 amino acids 1–222 & 254–337) rigid domain whose *RMSD*s are 0.6 and 1.6 Å, respectively. This segmentation is stable against variations in the rigidity threshold and does not require the execution the merging procedure. When we reduced the threshold to define the protein graph to 4.5 Å, we obtained two different domains: a small domain (amino acids 193–246) and a large domain (amino acids 1–192 and 247–337) whose RMSDs deteriorated to 2.7 and 3.0 Å, respectively.

To avoid duplication of features involving vertices and edges, we modify the construction of the line graph by discarding an edge if its two end vertices are connected as well. That way, features extracted from edges add new information. Finally, we use a merging routine with heuristic criteria to merge two domains. One may ask if we could skip the labeling step (Viterbi algorithm) and apply the merging routine directly to the clusters found by coarse graining. This simplified version of our algorithm achieves good results on proteins showing a large-scale movement, but fails on more subtle cases. Overall, post-processing via the merging procedure compensates for segmentation errors involving small fragments.

The running time of our algorithm depends on the size of the protein, the density of the protein graph, and the rigidity of the conformational change. Additional file 1: Figure S3 shows the relationship between protein size and the running time of our graph-based segmentation algorithm. We note that the running time for proteins smaller than 800 amino acids grows slowly in a linear fashion. For the larger proteins, it seems to grow quadratically. There are a few outlier proteins whose running time is significantly longer than for proteins of similar size.

Indeed, the running time strongly depends on how often the Viterbi algorithm is executed in the recursion and how quickly a big, non-rigid graph is segmented into several subgraphs. The worst scenario occurs when many Viterbi calculations are required for a protein with densely connected protein graph and with a high degree of flexibility such as intrinsically disordered proteins [26]. In these problematic cases, the signal derived from the mean-variance metric fails to distinguish the labels of inter/intra vertices and edges in the line graph.

Other segmentation methods and ours all require 3D protein structures which are not always available. In our graph-based framework, we may resolve this shortcoming by estimating a protein graph as follows. First, from a given protein primary sequence, we may use its protein contact map predicted, for example, by AlphaFold [27] to construct a protein graph. Second, due to the absence of 3D protein structures, the rigidity estimation could not base on RMSD but rather on another quantity which could be inferred directly from the protein contact map. Final, the rest of the graph-based method is unchanged and still applicable with above predicted protein graph.

## Conclusion

We present a new algorithm to characterize structural transitions in proteins. Our graph-based algorithm constructs a graph from a set of protein conformations and detects rigid domains via an edge labeling strategy. A key feature is that the number of rigid domains is determined automatically. Yet the algorithm allows users to relax the rigidity definition of domains and thereby increase or decrease the number of rigid domains. Segmentations produced by our algorithm agree strongly with segmentations found by other methods such as DynDom [3, 28] and Spectrus [6] on various medium to large scale structural transitions.

Our approach has several advantages over other rigid segmentation methods. First, there is no limitation on the number of protein conformations. In fact, a larger number of conformations should result in a better signal and thereby a superior performance of the algorithm. Second, by using the graph-based model along with a binary labeling of edges, we overcome the need to choose the number of rigid domains, which is necessary for many of the existing methods. Moreover, our method performs well with default parameter settings, which saves the user from parameter tweaking. Another appealing aspect of our method is that it can be used to produce a good initial segmentation for other segmentation algorithms. For instance, the $Nguyen \& Habeck2016$ method [8] requires a good initial guess of the rigid-domain segmentation which could be provided by our graph-based method. Finally, our graph-based framework is quite flexible in that it allows us to integrate into the scoring function additional information such as the location of hinges or a prior segmentation.

## Methods

We organize the “Methods” section as follows. First, we present the notation used throught the Methods section. Next, we describe several steps in our approach such as the coarse-graining algorithm used to reduce the graph size, a line graph transformation that enables inference of edges’ labels , and an outlier-detection method that we use to define features on the line graph. Moreover, we explain our method from the perspective of conditional random fields (CRFs) as well as our objective function for labellings of the line graph. Finally, we present pseudo code for our algorithm as well as a post-processing procedure.

### Notation

Our algorithm aims to infer a rigid-domain segmentation from $M>1$ conformational states of a protein. Each conformational state is encoded by a $N \times 3$ matrix $X\in {\mathbb {R}}^{N\times 3}$ whose rows are the 3D coordinates of representative atoms (typically $\mathrm {C}\alpha$ atoms), i.e. $X_{n}^{(m)}$ is the position of the *n*th atom in the *m*th conformation. Every conformational state gives rise to a symmetric $N\times N$ distance matrix $D^{(m)}$:

1 $$\begin{aligned} D^{(m)}_{k,l} := \Vert X_{k}^{(m)} - X_{l}^{(m)}\Vert \quad (k,l=1,2,\ldots , N), \end{aligned}$$

where $\Vert \cdot \Vert$ denotes the Euclidian norm.

We encode the conformational variability across all *M* structures through a *protein graph*

2 $$\begin{aligned} \mathcal {PG}=\left ({\mathcal {V}}, {\mathcal {E}}\right ) \end{aligned}$$

whose vertices ${\mathcal {V}}$ are the representative atoms $\left \{1,2,\ldots ,N\right \}$. An edge between atoms *k*, *l* belongs to the edge set ${\mathcal {E}}$ if and only if

3 $$\begin{aligned} \max _{m=1,2,\ldots ,M} D^{(m)}_{k,l} \le \delta \end{aligned}$$

where $\delta$ is a cutoff distance. Viloria et al. [29] suggest a cutoff distance of 5 Å as optimal value for molecular dynamics simulations. In contrast, HingeProt [9] uses 13 Å as a cutoff to construct a network. Our choice of the cutoff distance is inspired by elastic network models [30], which also encode protein structures as graphs. We ran tests with various cutoff values $\delta = 7.5$, 10.5 and 13.5 Å. We assess the rigidity of a subset ${\mathcal {S}}\subseteq {\mathcal {V}}$ through

4 $$\begin{aligned} {\text{RMSD}}_{}\left ({\mathcal {S}}\right ) := \frac{2}{M(M-1)} \sum _{m=1}^{M-1} \sum _{m'=m+1}^{M}{\text{RMSD}}_{{\mathcal {S}}}\left (X^{(m)},X^{(m')}\right ) \end{aligned}$$

where ${\text{RMSD}}_{{\mathcal {S}}}\left (X^{(m)},X^{(m')}\right )$ is the root mean square deviation (RMSD) [31] between conformations $X^{(m)}$ and $X^{(m')}$ reduced to atoms in ${\mathcal {S}}$. A subset ${\mathcal {S}}$ is rigid if and only if ${\text{RMSD}}_{}\left ({\mathcal {S}}\right ) < \theta$. The rigidity threshold $\theta$ depends on the heterogeneity of the conformational states. RigidFinder [5] probes every cutoff between 1.0 and 6.0 Å. We typically set $\theta =3.5$ Å in our tests on the DynDom benchmark [28].

### Coarse graining of the protein graph

Rigid domains form densely connected subsets of nodes in the protein graph. To reduce the size of the protein graph, we run the Louvain algorithm [13, 32, 33] that partitions the nodes ${\mathcal {V}}$ into communities. The parameters of the Louvain algorithm are chosen such that the communities

- are small enough to include, with a few exceptions, amino acids that are part of the same rigid domain (i.e. criterion (Eq. 4) is met for every community);
- are large enough to enable the inference of vertex labels (Eq. 9).

If ${\mathcal {C}}$ is a partition found by the Louvain algorithm, the *coarse-grained graph*

5 $$\begin{aligned} \mathcal {CG}=\left (\mathcal {CV}, \mathcal {CE}\right ) \end{aligned}$$

links two communities $c_1$ and $c_2$ ($c_1,c_2 \in {\mathcal {C}}$) by an undirected edge $(c_1,c_2)\in \mathcal {CE}$ if at least one pair of amino acids $a_1\in c_1, a_2\in c_2$ is linked in the protein graph: $(a_1,a_2)\in {\mathcal {E}}$. In this context, we use the expressions “vertex in the coarse-grained graph” and “community” interchangeably.

The mean variance of all distances between two communities $c_1$ and $c_2$ is defined by

6 $$\begin{aligned} \xi _{D}(c_1,c_2):= & {} \frac{1}{|c_1||c_2|(M-1)}\times \nonumber \\&\times \sum _{a_1\in c_1} \sum _{a_2\in c_2} \sum _{m=1}^M\left( D^{(m)}_{a_1,a_2}- \frac{1}{M}\sum _{m'=1}^M D^{(m')}_{a_1,a_2}\right) ^2\, . \end{aligned}$$

The mean variance is a key quantity of our method. For better readability we skip the subscript when it does not lead to misunderstandings.

We also use ${\text{RMSD}}_{}\left (\mathcal {CG}\right )$ to denote the root mean square deviation calculated from the protein graph of $\mathcal {CG}$ according to Eq. (4).

### Line graph transformation

Given an undirected graph with defined sets of vertices and edges, its line graph transformation is a graph whose vertices are the edges in the original graph [34]. Two vertices in the line graph are linked if and only if their corresponding edges in the original graph are incident (share a common vertex).

In this study, we apply the line graph transformation to the coarse-grained graph with a small modification. This transformation is an *intermediate* step that allows us to utilize the generalized Viterbi algorithm to infer binary labels of edges in the coarse-grained graph. The line graph derived from the coarse-grained graph is denoted as:

7 $$\begin{aligned} \mathcal {LG(CG)}=\left (\mathcal {LV}, \mathcal {LE}\right ) \end{aligned}$$

where the edges of the coarse-grained graph become the nodes of the line graph, or $\mathcal {LV} = \mathcal {CE}$. Two vertices are linked if and only if their two corresponding edges in the coarse-grained graph are incident and the two end nodes are not connected. Formally, we denote two adjacent vertices $v_1=(c_0, c_1)$ and $v_2=(c_0,c_2)$ where $v_1,v_2 \in \mathcal {LV}$, and $c_0,c_1,c_2 \in \mathcal {CV}$. In this notation, we call $c_0$ as a common vertex/node between $v_1$ and $v_2$, while $c_1, c_2$ are end nodes. We create an edge $e=(v_1,v_2) \in \mathcal {LE}$ if and only if $c_0$ is a common node and $(c_1,c_2) \notin \mathcal {CE}$.

Additionally, we define the mean variance of a vertex *v* in the line graph $\xi (v)$ according to Eq. (6) evaluated on both communities linked by *v*. Similarly, the mean variance of an edge *e* in the line graph is denoted by $\xi (e)$ and defined via the same equation applied to the end nodes of *e*.

### Outlier detection

The bigger the mean variance of a line graph vertex, the more likely is it that the corresponding communities belong to two different domains. Likewise, the end nodes of an edge tend to belong to different domains if the mean variance is large. However, it is not obvious how to define a mapping that is valid across a diverse set of proteins.

Motivated by these observations, we denote by an *inter/intra* vertex a line graph node linking two communities that are part of different domains/the same domain, respectively. Similarly, a line graph edged is an *inter* edge if its end nodes belong to different rigid domains; otherwise it is an intra-domain edge. We note that the mean variance of inter/intra vertices or edges follow two different but overlapping distributions. Both distributions can be modeled with inverse gamma distributions whose parameters can be estimated with expectation maximization (EM). However, we obtained very poor results with this approach due to the small number of inter vertices/edges. Therefore, we only consider the distribution of values from intra vertices/edges and treat values of inter vertices/edges as outliers.

To identify outliers, we use the algorithm developed by [35] that detects outliers based on the distance from its median normalized by the median absolute deviation (*MAD*) [36]. MAD is a measure of dispersion estimated via the median of absolute deviations from the median of the data. We consider a line graph ${\mathcal {G}}=({\mathcal {V}},{\mathcal {E}})$ with *P* vertices $v_i \in {\mathcal {V}}$ $(i=1\ldots P)$ and *Q* edges $e_j \in {\mathcal {E}}$ $(j=1\ldots Q)$. Without loss of generality, we enumerate the line graph vertices such that elements in the array of mean variances ${\mathcal {A}}_{vertex} = \left[ \xi (v_1),\xi (v_2),\ldots ,\xi (v_P)\right]$ are sorted in ascending order. Correspondingly, ${\mathcal {A}}_{edge}=\left[ \xi (e_1),\xi (e_2),\ldots ,\xi (e_Q)\right]$ is the array of mean variances of all edges indexed such that their mean variance increases. For both arrays, we define a binary outlier indicator $\gamma \in \{-1,+1\}$:

$$\begin{aligned} \gamma \left( v | {\mathcal {A}}_{vertex}\right) =\gamma _v:= {\left\{ \begin{array}{ll} -1 &{}\quad {\text {if }}v{\text { is an outlier in }}{\mathcal {A}}_{vertex};\\ +1 &{}\quad {\text {otherwise}}. \end{array}\right. } \end{aligned}$$

and

$$\begin{aligned} \gamma \left( e | {\mathcal {A}}_{edge}\right) =\gamma _e:= {\left\{ \begin{array}{ll} -1 &{} \quad {\text {if }}e{\text { is an outlier in }}{\mathcal {A}}_{edge};\\ +1 &{}\quad {\text {otherwise}}. \end{array}\right. } \end{aligned}$$

When the ascending mean variance arrays of vertices and edges are unambiguous in the given context, we omit the array and indicate whether we are considering vertex or edge arrays by the subscript.

Outliers are characterized by a mean variance that is larger than any other mean variance. The set of outliers can be enlarged by including non-outliers located at the end of the array. By such expanding, it is important to notice that the indices of outliers are always bigger than ones of non-outliers.

### A short introduction into CRFs

Let us consider a graph ${\mathcal {G}}=({\mathcal {V}},{\mathcal {E}})$ whose nodes we call *sites* and ${\mathcal {V}}= \{1,2,\ldots , N\}$ without loss of generality. Sites are labeled by elements of the finite set ${\mathcal {B}}$. Words of length $\ell$ over the finite alphabet ${\mathcal {O}}$ are called *observations*. ${\mathcal {E}}$ is the set of edges in the site graph ${\mathcal {G}}$. The neighborhood ${\mathcal {N}}_i\subseteq {\mathcal {V}}$ of site $i\in {\mathcal {V}}$ consists of all sites $j \in {\mathcal {V}}, j\not =i$ that are linked to *i* by an edge in ${\mathcal {N}}$ and $i\not \in {\mathcal {N}}_i$. For every label sequence ${\varvec{y}}\in {\mathcal {B}}^N$ and subset $I\subseteq {\mathcal {V}}$, ${\varvec{y}}_I$ denotes the partial labeling of sites in *I*: ${\varvec{y}}_I:=\{(i,y_i)\,|\, i\in I\}$. Additionally, for every $e \in {\mathcal {E}}$, ${\varvec{y}}_e$ denotes the labels of two vertices of *e* and ${\varvec{y}}_{{\mathcal {G}}^{'}}$ is the labels of all vertices in a graph ${\mathcal {G}}^{'}$.

A pair $({\varvec{X}},{\varvec{Y}})$ composed of a random observation ${\varvec{X}}\in {\mathcal {O}}^N$ and a random label sequence ${\varvec{Y}}\in {\mathcal {B}}^N$ realizes a feature-based exponential model if the conditional probability ${{\,\mathrm{p}\,}}\left( {\varvec{y}} \left| {\varvec{x}}\right. \right)$ of all pairs $({\varvec{x}},{\varvec{y}})$ is

8 $$\begin{aligned} {{\,\mathrm{p}\,}}\left( {\varvec{y}} \left| {\varvec{x}}\right. \right) = \frac{1}{Z({\varvec{x}})}\exp \left( \sum _{s=1}^c\sum _{|I|=s} \Psi ^{(s)}({\varvec{y}}_I,{\varvec{x}})\right) , \end{aligned}$$

where

$$\begin{aligned} Z({\varvec{x}}):= \sum _{{\varvec{y}}^\prime \in {\mathcal {B}}^N}\exp \left( \sum _{s=1}^c\sum _{|I|=s}\Psi ^{(s)}({\varvec{y}}_I^\prime ,{\varvec{x}})\right) . \end{aligned}$$

$\sum _{|I|=s}$ denotes a sum over all cliques *I* of size *s* in ${\mathcal {G}}$; *c* is the maximum clique size. For every clique size $s\le c$, the function $\Psi ^{(s)}({\varvec{y}}_I,{\varvec{x}})$ is the feature of cliques of size *s*. Under very weak assumptions the feature-based exponential models coincide with the class of *conditional random fields* where at every site *i* the label is conditionally independent of the labels outside ${\mathcal {N}}_i$ given the observation and the labels of ${\mathcal {N}}_i$.

The labeling problem is solved by computing a labeling sequence

9 $$\begin{aligned} {\varvec{y}}^* := \mathop {{{\,\mathrm{argmax}\,}}}\limits _{{\varvec{y}}\in {\mathcal {B}}^N}{{\,\mathrm{p}\,}}\left( {\varvec{y}} \left| {\varvec{x}}\right. \right) \end{aligned}$$

that achieves maximum posterior probability (MAP prediction). In general, MAP prediction is $\mathbf {NP}$-hard. The generalized Viterbi algorithm detailed in [14] is able to make the inference for an arbitrary graph, yet has an exponential running time according to the boundary set of a graph. Only if the underlying site graph is small enough, it can be used within a feasible time bound.

#### Label inference via the generalized Viterbi algorithm

A shortcoming of existing rigid-domain detection methods such as [6, 8, 28] is the requirement to specify the number of rigid domains which is often unknown. To overcome this issue, we use the generalized Viterbi algorithm to infer a binary labeling which indicates if a pair of nodes in the coarse-grained graph belongs to identical or different rigid domains. It is important to note that we need to infer the binary labels of *edges* in the coarse-grained graph, whereas the Viterbi algorithm estimates optimal *vertex* labels. Thus, it is not suitable to directly apply the Viterbi algorithm to the coarse-grained graph. Instead, we apply the generalized Viterbi algorithm on a line graph derived from the coarse-grained graph. This gives us a binary labeling of line graph vertices, which equivalent to a binary labeling of edges in the coarse-grained graph.

Thus, we consider a line graph as a site graph described above. In a pairwise CRF, one only considers cliques formed by vertices and edges. Consequently, Eq. (8) can be rewritten as

10 $$\begin{aligned} {{\,\mathrm{p}\,}}\left( {\varvec{y}} \left| {\mathcal {V}},{\mathcal {E}}\right. \right) \sim \exp \left( \sum _{v \in {\mathcal {V}}} \Psi ^{(1)}(v,{\varvec{y}}_v) + \sum _{(v_1,v_2) \in {\mathcal {E}}} \Psi ^{(2)}(v_1,v_2,{\varvec{y}}_{(v_1,v_2)}) \right) \end{aligned}$$

where $\Psi ^{(1)}$ and $\Psi ^{(2)}$ are the feature functions defined on vertices and edges respectively. The term $Z({\varvec{x}})$ can be ignored because it is not a function of ${\varvec{y}}$. As a convention, we call ${{\,\mathrm{p}\,}}\left( {\varvec{y}} \left| {\mathcal {V}},{\mathcal {E}}\right. \right)$ “*unnormalized probability*” or “*scoring function*” interchangeably.

In our rigid domains detection problem, we define a feature function for a vertex *v* along with its label ${\varvec{y}}_v$ by

11 $$\begin{aligned} \Psi ^{(1)}(v,{\varvec{y}}_v) = \gamma _v{\varvec{y}}_v\, . \end{aligned}$$

This function will reward labeling ${\varvec{y}}_v$ that coincide with the outlier indicator value.

Given an edge $e=(v_1,v_2) \in {\mathcal {E}}$, we define a feature function on *e* and its predicted label ${\varvec{y}}_e$ by distinguishing three cases:

*Case “Two values among* $\gamma _{e}, \gamma _{v_1}, \gamma _{v_2}$ *are equal to* $-1$.” In this case, the egde feature rewards an agreement between the predicted vertex labels ${\varvec{y}}_e$ and the outlier indicators:

12 $$\begin{aligned} \Psi ^{(2)}\left( e,{\varvec{y}}_e | e=(v_1,v_2)\right) := {\left\{ \begin{array}{ll} +1 &{} \, {\text {if }}{\varvec{y}}_{v_1}\gamma _{v_1} + {\varvec{y}}_{v_2}\gamma {v_2} = 2;\\ -1 &{}\, {\text {otherwise}}. \end{array}\right. } \end{aligned}$$

*Case* “$\gamma _{v_1}=\gamma _{v_2}=+1$” seems to indicate that three nodes of $v_1$ and $v_2$ (a common vertex and two end nodes) belong to the same rigid component. However, the vertex shared by the two edges may be part of a hinge region between two rigid components. This is likely to occur if the mean variance value of the edge is outlier, or “$\gamma _e=-1$”. If this is the case, we have to decide to which component the hinge node belongs. This decision is based on a comparison between $\xi (v_1)$ and $\xi (v_2)$. Thus, $\Psi ^{(2)}$ becomes:

13 $$\begin{aligned}&\Psi ^{(2)}\left( e,{\varvec{y}}_e | e=(v_1,v_2)\right) \nonumber \\&\quad :={\left\{ \begin{array}{ll} +1 &{} \quad {\text {if }}{\varvec{y}}_{v_1}=-1, {\varvec{y}}_{v_2}=+1, \gamma _e=-1\text { and }\xi _{v_1}>\xi _{v_2};\\ +1 &{} \quad {\text {if }}{\varvec{y}}_{v_1}=+1, {\varvec{y}}_{v_2}=-1, \gamma _e=-1\text { and }\xi _{v_1}<\xi _{v_2};\\ +1 &{} \quad {\text {if }}{\varvec{y}}_{v_1}={\varvec{y}}_{v_2}=+1\text { and }\gamma _e=+1;\\ 0 &{} \quad {\text {if }}{\varvec{y}}_{v_1} {\varvec{y}}_{v_2}=-1, \gamma _e=-1\text { and }\xi _{v_1}=\xi _{v_2};\\ -1 &{}\quad {\text {otherwise}}. \end{array}\right. } \end{aligned}$$

*For any other combination of* $\gamma _{v_1}, \gamma _{v_2}$ *and* $\gamma _e$, we set

14 $$\begin{aligned} \Psi ^{(2)}\left( e,{\varvec{y}}_e \right) := 0 \end{aligned}$$

In all three cases above, labelings are rewarded by setting $\Psi ^{(2)}$ to $+1$, penalized by setting $\Psi ^{(2)}$ to $-1$ and ignored by setting $\Psi ^{(2)}$ to 0.

Hence, for any labeling of the line graph ${\mathcal {G}}$, the generalized Viterbi algorithm computes its unnormalized probability (Eq.  10) via Eqs. (11)–(14) and thus gives us the most probable labels of ${\mathcal {G}}$.

### Graph-based prediction of rigid domains

This subsection provides pseudo code for our graph-based prediction of rigid domains in proteins. We denote the rigidity threshold as $\theta$ (typically 3.5 Å). 
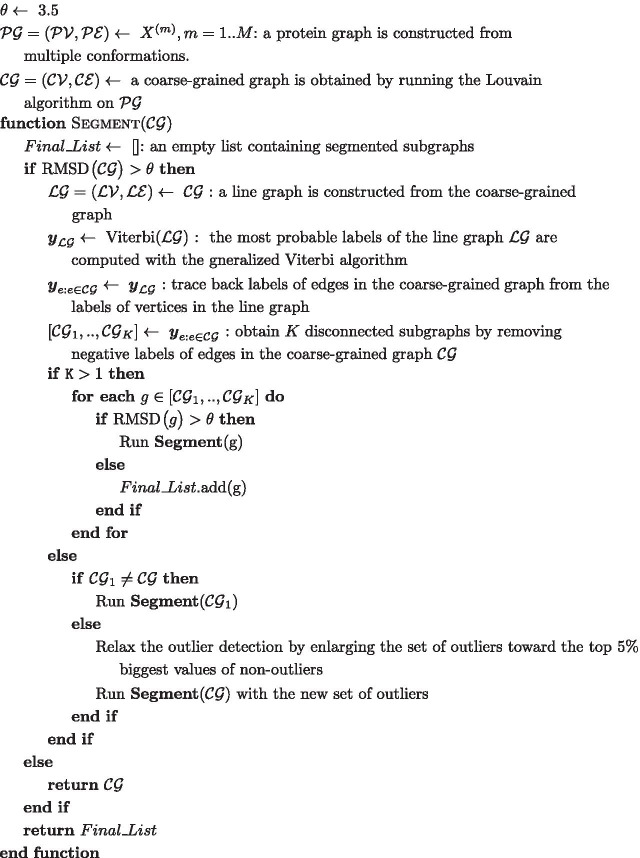


There is no guarantee that this algorithm always converges. However, we experienced fast convergence within a few iterations in most of our experiments. We also added a limitation on the number of recursions. The final result of our algorithm is a list of disconnected subgraphs of the coarse-grained graph.

### Finalizing rigid-domain segmentation

Our graph-based method for rigid-domain detection described in the Sect. 5.6 produces a list of disconnected subgraphs of the reduced graph. we can trace back the subgraphs to the corresponding protein subgraphs and thus obtain a list of disconnected protein graphs.

Let ${\mathcal {S}}=\{{\mathcal {S}}_1,{\mathcal {S}}_2,\ldots ,{\mathcal {S}}_L\}$ be a mutual exclusive partition of the protein graph $\mathcal {PG}$. Our merging algorithm works as follows: 
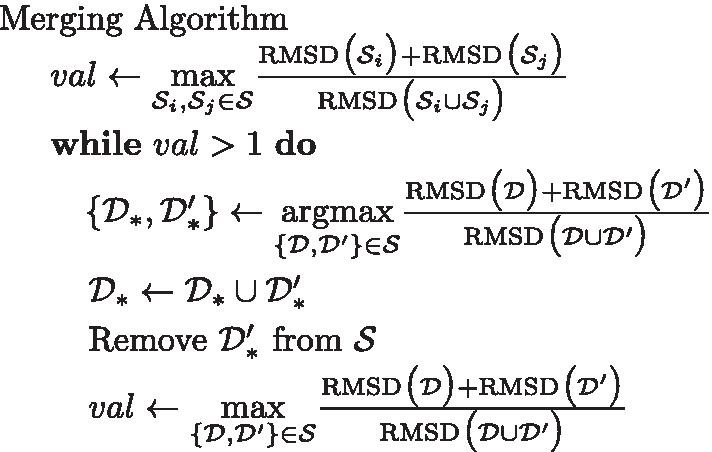


After termination of the Merging Algorithm, ${\mathcal {S}}$ is returned as rigid-domain prediction.

## Supplementary information

**Additional file 1:** The support information includes the follows. The first section is the analyses of inconsistency error of reduced graph. The second section is the analyses of signal enhancement by coarse graining. The third section contains PDB codes of Lysozyme protein. The fourth section is the analyses of the running time.

## Data Availability

Web-server: http://azifi.tz.agrar.uni-goettingen.de/webservice/ Source code: https://github.com/dtklinh/GBRDE.
