# Supplementary material for: A graph-based algorithm for detecting rigid domains in protein structures
Source: BMC Bioinformatics. 2021 Feb 12;22:66. doi: 10.1186/s12859-021-03966-3 (PMC7881620; doi:10.1186/s12859-021-03966-3)
Supplement: Supplementary file 1 — Additional file 1: The support information includes the follows. The first section is the analyses of inconsistency error of reduced graph. The second section is the analyses of signal enhancement by coarse graining. The third section contains PDB codes of Lysozyme protein. The fourth section is the analyses of the running time. [file 12859_2021_3966_MOESM1_ESM.pdf]

1 Inconsistency error of reduced graph

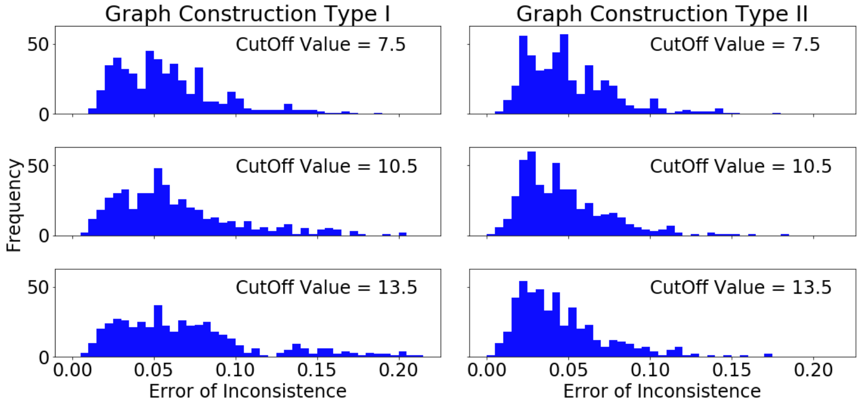

Figure S1: Histogram of inconsistency error from graph construction type I and II and their varying cutoff values respectively.

2 Signal enhancement by coarse graining

As in the Methods and Discussion sections, let $\mathcal{G} = (\mathcal{V}, \mathcal{E})$ denote a graph and $\mathcal{LG} = (\mathcal{LV}, \mathcal{LE})$ the line graph derived from $\mathcal{G}$. For each vertex $v \equiv (v_0, v_1) \in \mathcal{LV}$ $(v_0, v_1 \in \mathcal{V})$, $\xi(v) = \xi(v_0, v_1)$ is the mean-variance of v according to the Equation (6) in the Methods section. The label of v is $\sigma_v = +1$ (intra-vertex) if $\sigma_{v_0} = \sigma_{v_1}$ and -1 (inter-vertex) otherwise. For each edge $e \equiv (v_l, v_m, v_r) \in \mathcal{LE}$ $(v_l, v_m, v_r \in \mathcal{V}; (v_l, v_m), (v_m, v_r) \in \mathcal{E}$ & $(v_l, v_r) \notin \mathcal{E})$, its mean-variance $\xi(e) \equiv \xi(v_l, v_r)$ is calculated as above. The label of an edge in the line graph is $\sigma_{e^*} = +1$ (intra-edge) if $\sigma_{v_l} = \sigma_{v_r}$, and -1 (inter-edge) otherwise.

The performance of the CRF scoring function depends on how well the values of mean-variance of inter and intra-vertices/edges separate in the line graph. We assess this separability by the mean of the area under the ROC curve (AUC) through the line graphs derived from the protein graph and from the coarse-grained graph.

With the thresholding of the mean-variance as a classifier, the Panels S2.A and S2.B show that inter and intra-vertices/edges in the line graph derived from the coarse-grained graph significantly achieve bigger separability than the ones in the line graph derived from the protein graph. Therefore, the coarse-graining process does not only reduce the graph size, but it also enriches the information represented by the mean-variance.

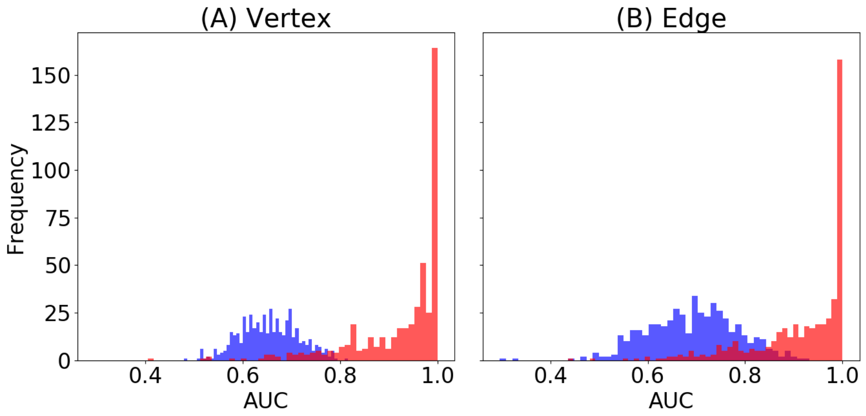

Figure S2: Histograms of area under the ROC curve (AUC) evaluated on 487 proteins in the DynDom dataset. (A) Histograms of AUC calculated from the inter and intra-vertices in the line graphs derived from the protein graph(blue histogram) and from the coarse-grained graph (red histogram). (B) Histograms of AUC calculated from the inter and intra-edges in the line graphs derived from the protein graph(blue histogram) and from the coarse-grained graph (red histogram).

3 Lysozyme protein

PDB codes used in the study

150L_D 173L_A 150L_A 150L_C 150L_B 3SB8_C 3GUN_A 3GUN_B 3GUL_B 3GUL_A 152L_A 2HUK_A 2Q9E_C 4S0W_A 1JQU_D 1JQU_A 1JQU_B 1JQU_C 149L_A 145L_A 151L_A 2HUM_B 2HUM_A 3GUK_A 3GUK_B 137L_B 3FI5_A 3SB9_B 1L97_A 1L97_B 3GUI_A 178L_A 3GUO_A 3GUO_B 201L_B 201L_A 3GUM_B 3GUM_A 168L_B 168L_C 168L_A 168L_D 168L_E 172L_A 3GUJ_A 209L_A 1P7S_A 1KNI_A 1SSY_B 1SSY_A 1QTH_B 4PK0_A 4UIS_G 169L_A 169L_C 169L_B 169L_E 169L_D 4GBR_B 1LWG_A 3GUP_B 167L_B 3GUP_A 2LCB_A 3SB9_A 3SBA_F 3SBA_D 3SBA_E 3SBA_B 3SBA_C 3SBA_A 2QAR_C 2QAR_F 1PQK_C 4PJZ_A 171L_A 3SBB_C 218L_A 3SB7_A 3SB7_B 1QTH_A 3JR6_A 3JR6_B 3JR6_C 3JR6_D 3SB5_B 3SB5_C 3SB5_A 3SB5_D 214L_A 1T8G_A 189L_A 1P5C_A 1P5C_B 1P5C_C 1P5C_D 216L_B 216L_A 174L_B 174L_A

4 Running time

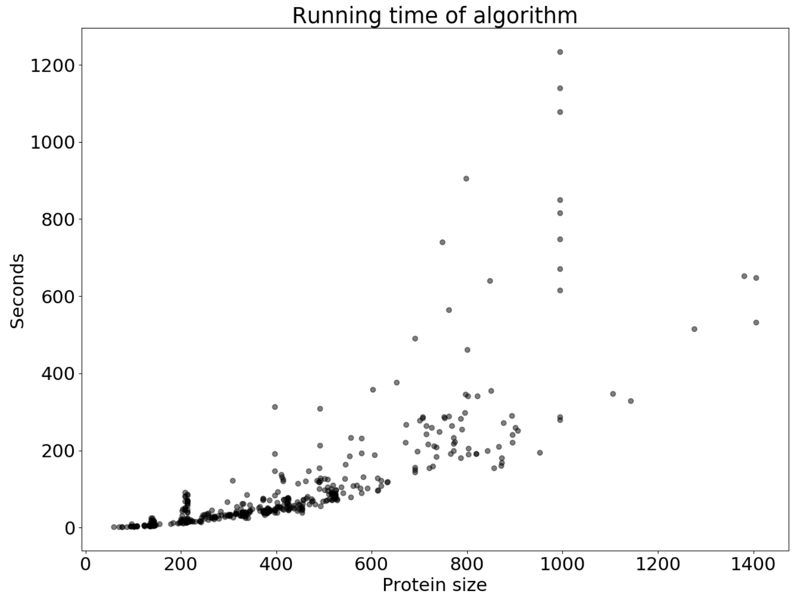

Figure S3: Protein size versus running time (measured in seconds) evaluated for 487 proteins selected from the DynDom database.
